# O-11 - EXAMINING THE ASSOCIATION BETWEEN SENTINEL RESPIRATORY SURVEILLANCE AND ANTIBIOTIC PRESCRIBING IN SCOTLAND: ANALYSIS OF INDIVIDUAL-LEVEL LINKED DATA

**DOI:** 10.1093/pubmed/fdag046.011

**Published:** 2026-07-09

**Authors:** Dr Ronan McCabe, Dr Helen Gadegaard, Dr Kimberly Marsh, Dr Josie Evans

**Affiliations:** Public Health Scotland; Public Health Scotland; Public Health Scotland; Public Health Scotland

## Abstract

**Background:**

GP practices registered to Scotland’s Community Acute Respiratory Infection (CARI) sentinel surveillance programme have reported that access to CARI’s 10 pathogen (predominantly viral) respiratory testing panel, with results available within 7-10 days, supports antimicrobial stewardship by helping ensure that patients with ARI receive the most clinically appropriate treatment.

**Objectives:**

We examined this association using individual-level linked data.

**Methods:**

We identified individuals recruited to CARI in the 2024-25 season. Our comparator population included individuals with a record of an ARI consultation in digital primary care records of CARI-registered practices. We linked individual-level data on prescriptions, demographics and test results, and used binary logistic regression to compare the adjusted odds of being prescribed an antibiotic among individuals within CARI, with those with an ARI consultation but not recruited into CARI. All models were adjusted for age, sex, deprivation and time (month).

**Results:**

There were 22,867 individuals within CARI and 47,525 in the comparator population. The odds of prescription on the day of consultation were 52.7% lower for those within CARI [aOR=0.473, 95%CI: 0.430 to 0.516]. This association remained for prescriptions within 14-days [aOR=0.778, 95%CI: 0.721 to 0.835] and 28-days [aOR=0.673, 95%CI: 0.625 to 0.721] of consultation, excluding the day of consultation.

**Conclusion:**

People with ARI recruited to CARI had lower odds of antibiotic prescription than those not recruited. This supports anecdotal reports that GPs use CARI recruitment to help tailor prescribing. However, the result could also be explained by unmeasured confounding or selection bias, if i) GPs are less likely to recruit to CARI when bacterial infection is suspected or ii) the comparator population of ARI GP consultations (which are known to be under-reported) are not a representative subset. Further investigation into why and how GPs recruit to CARI is warranted to understand this association.

